# Nucleation of Fe-rich phosphates and carbonates on microbial cells and exopolymeric substances

**DOI:** 10.3389/fmicb.2015.01024

**Published:** 2015-09-22

**Authors:** Mónica Sánchez-Román, Fernando Puente-Sánchez, Víctor Parro, Ricardo Amils

**Affiliations:** ^1^Department of Planetology and Habitability, Centro de Astrobiología (INTA-CSIC)Madrid, Spain; ^2^Department of Molecular Evolution, Centro de Astrobiología (INTA-CSIC)Madrid, Spain; ^3^Department of Virology and Microbiology, Centro de Biología Molecular Severo OchoaMadrid, Spain

**Keywords:** microbial, bacterial precipitates, nanoglobules, Tessarococcus, vivianite, siderite

## Abstract

Although phosphate and carbonate are important constituents in ancient and modern environments, it is not yet clear their biogeochemical relationships and their mechanisms of formation. Microbially mediated carbonate formation has been widely studied whereas little is known about the formation of phosphate minerals. Here we report that a new bacterial strain, *Tessarococcus lapidicaptus*, isolated from the subsurface of Rio Tinto basin (Huelva, SW Spain), is capable of precipitating Fe-rich phosphate and carbonate minerals. We observed morphological differences between phosphate and carbonate, which may help us to recognize these minerals in terrestrial and extraterrestrial environments. Finally, considering the scarcity and the unequal distribution and preservation patterns of phosphate and carbonates, respectively, in the geological record and the biomineralization process that produces those minerals, we propose a hypothesis for the lack of Fe-phosphates in natural environments and ancient rocks.

## Introduction

*Authigenic* ferrous iron-rich minerals like vivianite [Fe_3_(PO_4_)_2_ × 8H_2_O] and siderite (Fe_2_CO_3_) are used as indicators of paleoenvironmental conditions, diagenetic evolution of sedimentary sequences (Last and De Deckker, 1990; Manning et al., 1999; Sapota et al., 2006) and biosignatures (Vuillemin et al., 2013; Sánchez-Román et al., 2014). They are usually found associated in organic rich environments like lacustrine (Lemos et al., 2007; Rothe et al., 2014) and deep-sea sediments (Dijkstra et al., 2014), swamps, sewage, and wastewater treatment plants (Postma, 1981; Lovley et al., 1991). Vivianite is considered the most important sink of phosphorus in reducing natural systems, being a significant parameter controlling the trophic status of lakes (Nriagu and Dell, 1974; Manning et al., 1991). Therefore, it can exert significant controls over the geochemical cycles of P and Fe (Veeramani et al., 2011) in reducing sediments in which iron and phosphorous are highly mobile and the sulfide ion is not produced in high concentration (Manning et al., 1991). On the other hand, the majority of the carbonate minerals on Earth surface are of biogenic origin (Moore, 1989; Riding, 2006) and the process of carbonate precipitation can be the most important factor controlling the global carbon cycling (Ridgwell and Zeebe, 2005; Dupraz et al., 2009). Vivianite and siderite usually occur associated with pyrite (FeS_2_) in veins of copper, tin, iron, and gold ores (Craig and Vaughan, 1994; Wiberg et al., 2001). Furthermore, these two iron-rich minerals are used as iron fertilizer (Eynard et al., 1992; Rakshit et al., 2008; Sánchez-Alcalá et al., 2012) and more rarely vivianite has been used as phosphorous fertilizer (Mikhailov, 1940; Nelipa, 1961). In addition, vivianite is also found in decaying plants and animal tissues, bones, shells, anthropogenic compounds, human wastes, and archeological settings (Jakobsen, 1988; McGowan and Prangnell, 2006; Nutt and Swihart, 2012).

Vivianite is a significant mineral because links forensic medicine (Thali et al., 2011), physical anthropology (McGowan and Prangnell, 2006), biology and climate geology (Sapota et al., 2006). On the other hand, siderite is also an important carbonate mineral that provides information about past climatic events on Earth and Mars (Ellwood et al., 1998; Fairen et al., 2004; Tomkinson et al., 2013). Actually, siderite and vivianite are significant constituents of martian meteorites and Mars surface (Valley et al., 1997; Dyar et al., 2014). In contrast to vivianite, siderite is found in much greater abundance in ancient rocks than in modern environments (Ohmoto et al., 2004; Kholodov and Butuzova, 2008). The formation of these two iron rich minerals is generally attributed to the activity of iron reducing bacteria (Mortimer and Coleman, 1997; Orange et al., 2009; Lee et al., 2010), and they have significant implications for microbial metabolism in sediments (Fredrickson et al., 1998). It is known that siderite can be formed within the sub-oxic, sulfate-reducion and methanogenic biogeochemical zones within the sediment column (Wilkinson et al., 2000).

Recently, Fe-rich sulfide (pyrite), sulfate [jarosite, KFe_3_(OH)_6_(SO_4_)_2_], and carbonate (siderite) minerals have been found in Rio Tinto basin (Fernández-Remolar et al., 2012) and their formation have been also related to iron-reducing fungi and bacteria (Oggerin et al., 2013; Sánchez-Román et al., 2014). Those minerals together with vivianite are known as important minerals in the iron biogeochemical cycle (Raiswell and Canfield, 2012). Rio Tinto is an acidic system in which microorganisms play an important role by determining the speciation of iron and can also cause considerable iron accumulation through biomineralization (Fernández-Remolar et al., 2012; Oggerin et al., 2013; Sánchez-Román et al., 2014). Furthermore, this acid-sulfate system enriched in iron is considered one of the potential analogs for early life on Earth and Mars (Fernández-Remolar et al., 2012). In order to better understand the nucleation and formation processes of iron carbonate and phosphate minerals, here, we present for the first time microbially mediated primary precipitation of siderite and vivianite in anaerobic culture experiments under Earth's surface conditions using a bacterial strain, *Tessarococcus lapidicaptus*, isolated from the subsurface of Rio Tinto (Puente-Sánchez et al., 2014a). The nucleation, chemical composition, texture and morphology of the bioprecipitates have been studied using a combination of high resolution transmission electron microscopy (TEM), scanning electron microscopy (SEM), sensitive energy dispersive X-ray Spectroscopy (EDS), and X-ray powder diffraction (XRD). We demonstrate that *T. lapidicaptus* produces spatially restricted supersaturated conditions and can overcome kinetic barrier to nucleate phosphate and carbonate nanocrystals in its cells and secreted EPS, respectively. We propose that microbial nanostructures, nanocrystals, and crystalline nanoparticles, are not related to a single microbial group or to a specific microbial metabolism but to a wide range of microorganisms. Finally, we discuss the mechanism of formation of both phosphate and carbonate and their significance and implication in natural systems.

## Materials and methods

### Microorganism

*Tessaracoccus lapidicaptus* CECT 8385 (= DSM 27266) is a gram-positive, non-spore forming, oval to rod shaped, nitrate-reducing, and facultatively anaerobic Actinobacterium. The growth temperature ranges from 15 to 40°C (optimal at 37°C) and the growth pH range from 6 to 9 (optimal at 8). It was isolated from a 297 m depth-drilling core obtained from the Iberian Pyrite Belt (Puente-Sánchez et al., 2014a). Only the innermost part of the core was sampled, and sodium bromide was added to the drilling water as a tracer for potential contamination, as described in Amils et al. (2013).

Two members of the *Tessaracoccus* genus have been previously isolated from marine sediments (Lee and Lee, 2008) and deep subsurface environments (Finster et al., 2009). Others have been isolated from crude oil-contaminated saline soil (Cai et al., 2011) and oleaginous, water-mixed metalworking fluids (Kämpfer et al., 2009), which suggests that the *Tessaracoccus* genus might be specially adept at degrading hydrocarbons and/or recalcitrant organic matter under harsh environmental conditions.

### Culture medium

The composition of the anoxic medium FE used in this study was (wt/vol): 0.25% NaCl; 0.04% NH_4_Cl; 0.003% MgCl_2_· 6H_2_O; 0.005% CaCl_2_· 2H_2_O; 0.2% FeCl_2_· 4H_2_0; 0.01% yeast extract; 0.085% NaNO_3_; 0.1% glucose; 0.1% succinic anhidryde; 0.05% KH_2_PO_4_; 0.025% NaHCO_3_; 0.05% cysteine hydrochloride; 0.01% resazurin. The pH of the medium was 6 and it was sterilized at 121°C for 20 min.

### Study of crystal nucleation and precipitation

*T. lapidicaptus* was inoculated into liquid cultures which were carried out in 100 ml bottles containing 100 ml of FE medium. The bottles containing the culture medium were incubated anaerobically at 30°C and examined periodically for the presence of minerals for up to 45 days after incubation. Controls consisting of uninoculated culture media and media inoculated with non-viable cells were included in all the experiments. pH measurements were performed at the end of the growth and mineral formation.

The optical density (OD) of the inocula was 0.5 at a wavelength of 600 nm. It was analyzed using a Spectronic 20 Genesys spectrophotometer. The Fe^2+^ was measured using a RQflex 10 Merck reflectoquant.

### Mineral analysis

The crystals were examined by X-ray diffraction (XRD) using a PANlytical X'Pert MPD PW3011/10. A JEOL JSM 6335 scanning electron microscope (SEM), equipped with a spectroscope of dispersive energy (EDX), was used for imaging and elemental analysis of single crystals. The mineral precipitates were also analyzed by transmission electro microscopy (TEM) under JEOL JEM 2100, 200 KV TEM with a CCD camera model 832. The morphology of the cells and crystal precipitates were examined with a JEOL JEM-1010 (TEM). TEM sample preparation is described in Ferrero et al. (2013) but without adding 5% K_3_Fe(CN)_6_ in the post-fixation step.

### Geochemical studies

The activity of dissolved species and the degree of saturation in the solutions assayed were determined using the geochemical computer program PHREEQC version 2 (Parkhust and Appelo, 1999). The results from PHREEQC are presented in terms of the saturation index (SI) for each predicted mineral. SI is defined by SI = lg (IAP/Ksp), where IAP is the ion activity product of the dissolved mineral constituents in a solubility product (Ksp) for the mineral. Thus, SI > 0 implies oversaturation with respect to the mineral, whereas SI < 0 means undersaturation.

## Results

**Figure 1:**
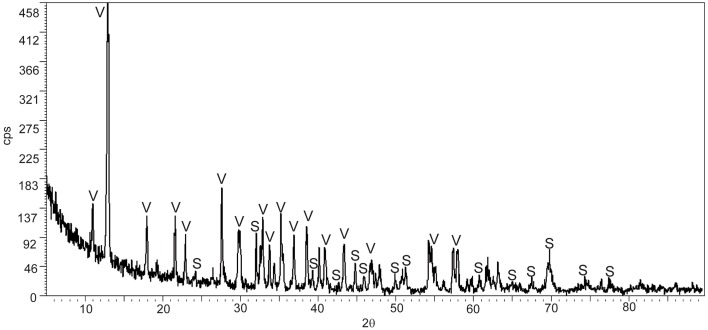
**X-ray diffractogram of the bioprecipitates formed in *T. Lapidicaptus* anaerobic cultures**. V, Vivianite; S, siderite.

The mineral precipitates formed exclusively in culture bottles with active bacterial cells, while no mineral precipitation occurred in sterile parallel controls (bottles with non-viable cells and without cells). The pH changed from 6 to ~7.5 in cultures with living bacteria. No change in pH was detected in the control experiments. The starting concentration of Fe^2+^ was 0.56 g/L and the final concentration, after mineral precipitation, 0.01 g/L. The XRD study reveals that the bioprecipitates are composed of vivianite and siderite, being vivianite the dominant mineral phase (Figure 1).

**Figure 2:**
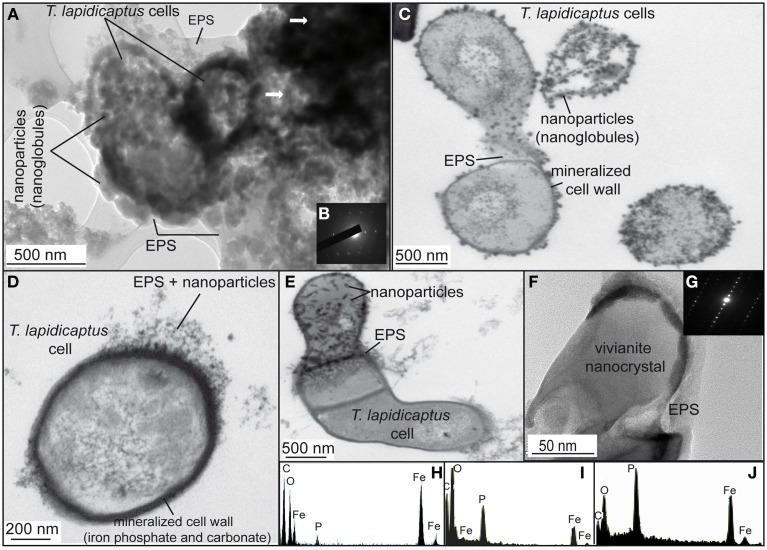
**TEM images of the bioprecipitates formed in *T. Lapidicaptus* anaerobic cultures**. **(A)** The bioprecipitates are nanoparticles attached to *T. lapidicaptus* cell and its secreted EPS, respectively. **(B)** Electron diffraction pattern of the darker areas, more mineralized areas. **(C)** *T. lapidicaptus* cells with mineralized cell wall and covered by nanoparticles embeded in EPS. **(D)** Detail of *T. lapidicaptus* cell with mineralized cell wall. Note nanoparticles embedded in EPS. **(E)** Detail of three cells together with mineralized cell wall and EPS. The upper cell covered by nanoparticles embedded in EPS. **(F)** Elongated nanoparticle, vivianite nanocrystal, embedded in EPS. **(G)** Electron diffraction pattern of the nanocrystal **(F)**. **(H,I)** EDX spectra of both dark and lighter mineralized areas (1A) composed of Fe-carbonate and phosphate, respectively. **(J)** EDX spectrum of nanoparticle (1E) composed of Fe-phosphate (vivianite).

**Figure 3:**
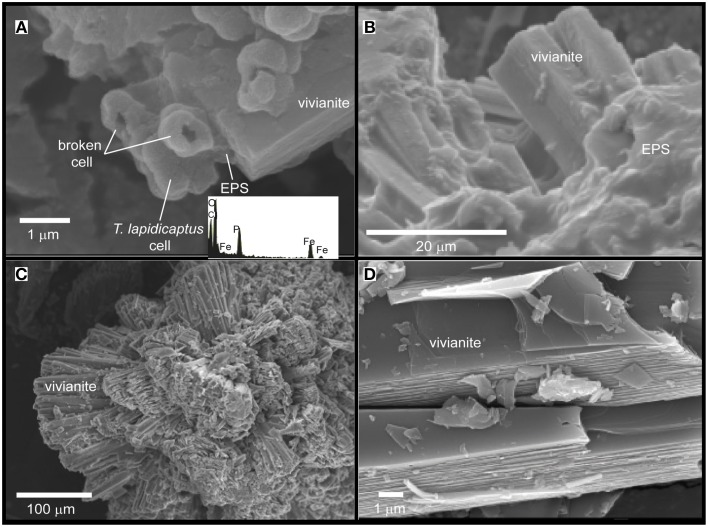
**SEM images of the Fe-phosphate precipitates from *T. Lapidicaptus* anaerobic cultures**. EDX spectrum of mineralized cell displaying C, O, Fe, and P. **(A)** Vivianite crystal attached to mineralized *T. lapidicaptus* cells and EPS. **(B)** Elongated vivianite crystal embedded in EPS. **(C)** Rosette formation of crystal clusters of vivianite. **(D)** Vivianite crystals with prismatic or tabular habit.

**Figure 4:**
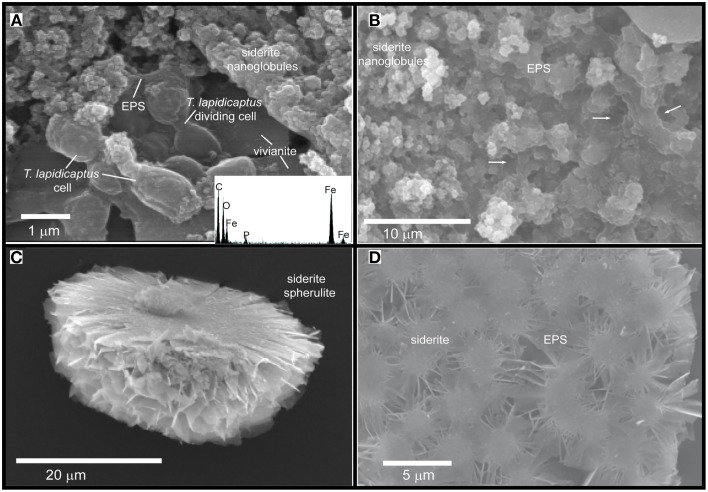
**SEM images of the Fe-carbonate precipitates from *T. Lapidicaptus* anaerobic cultures**. **(A)** Siderite nanoglobules embedded in EPS and attached to mineralized dividing *T. Lapidicaptus* cells. Note the vivianite crystal attached to these cells. EDX spectrum of mineralized cell displaying C, O, Fe, and small peak of P. **(B)** Fe-carbonate nanoglobules (siderite) embedded in EPS and delimiting the bacterial cell contours (white arrows). These nanostructures display granulated texture. White arrows correspond to moulds of degraded bacteria (broken cells). **(C)** Broken microspherulite of siderite. **(D)** Detail of a siderite spherulite which formed by aggregation of nanoparticles.

TEM and SEM images of the bacterial precipitates show that Fe-phosphate crystals and Fe-carbonate spheroidal nanoparticles (nanoglobules) and in some cases, elongated nanoparticles were attached to the bacterial cells and EPS (Figures 2A–D, 3A,B,D, 4A,B). EDX analyses (Figures 2F–H) confirm the X-ray results, the nanoparticle precipitates are composed of both, vivianite and siderite. Vivianite crystals have a prismatic or tabular habit and form coarse radial-fibrous aggregates like rosettes with a high degree of crystallinity and vitreous luster (Figures 3C,D). These crystals are approximately 10–20 μm in width and 100–300 μm in length. Siderite crystals are aggregates of nanoglobules with a diameter 20–100 nm (Figures 4A,B). These nanoglobules were attached to *T. lapidicaptus* cells and embedded in a thin organic film (exopolymeric substances or EPS) produced by *T. lapidicaptus* during its growth (Figures 2A–D, 4A,B). Mineralized bacteria were clearly recognized (Figures 3A, 4A) as well as dividing cells (Figures 2B, 4A); broken cells and mould of degraded cells (Figures 3A, 4B). The process of microspherulites (diameter > 10 μm) formation comprises a sequence of events, starting with the appearance of bacterial nanoglobules (<20 nm) to larger ones (>100 nm), which agglomerate with time resulting in microspherulites (Figures 4C,D). The most important process in the sequence that leads to the formation of spherulites is the accumulation of nanoglubules and mineralized bacterial cells, embedded in EPS matrix, displaying a granulated texture (Figures 4A,B,D).

Mineral phases with SI values positive or very close to 0 (above or below the equilibrium point) were observed, suggesting the possibility for inorganic (chemical) precipitation in the aqueous medium assayed (Table 1). These SI data were obtained by applying the geochemical software PHREEQC to the ionic composition of the culture medium. According to these data, the culture FE medium is saturated in hydroxiapatite, vivianite, and siderite.

**Table 1:** **Saturation index values (SI) in FE anaerobic medium**.

**Mineral phase**	**SI**
Aragonite, CaCO_3_	−1.16
Artinite, Mg_2_(CO_3_)(OH)_2_ × 3H_2_O	−8.45
Brucite, Mg(OH)_2_	−6.76
Calcite, CaCO_3_	−1.02
Dolomite, CaMg(CO_3_)_2_	−1.34
Halite, NaCl	−4.61
Huntite, CaMg_3_(CO_3_)_4_	−5.98
Hydroxiapatite Ca_5_(PO_4_)_3_OH	2.48
Magnesite, MgCO_3_	−0.81
Natron, Na_2_CO_3_× 10H_2_O	−7.46
Nesquehonite, MgCO_3_× 3H_2_O	−3.22
Siderite, FeCO_3_	2.40
Vivianite, Fe_3_(PO_4_)_2_× 8H_2_O	9.08

*Results are from geochemical software PHREEQC. SI values are for initial conditions*.

## Discussion

### Nucleation and precipitation of phosphate and carbonate by *T. lapidicaptus*

Our TEM and SEM studies showed that carbonate and phosphate nanocrystals nucleated on bacterial cell surfaces and EPS (Figures 2, 3, 4). The initial step of nucleation of carbonate and phosphate spheroidal nanocrystals occurs in the outer side of the bacterial envelopes (cell wall) and within EPS in intimate association with the bacteria cell surface (Figures 2A–D, 3A, 4A,B). Later carbonate spherulites and elongated phosphate crystals are formed by aggregation of nanocrystals embedded in the EPS matrix (Figures 2E, 3A,B, 4A,B,D). We also observed mineralized bacterial cells embedded in the surface of the crystals (Figures 3A, 4A). Similar nanocrystals have been previously reported for culture experiments using sulfate reducing-bacteria, aerobic heterotrophic bacteria, acidophilic iron-reducing bacteria and fungi (Aloisi et al., 2006; Bontognali et al., 2008; Sánchez-Román et al., 2008, 2014; Oggerin et al., 2013). These findings lead us to propose that microbial nanostructures such as nanocrystals and crystalline nanoparticles are not related to a single microbial group or to a specific microbial metabolism but to a wide range of microorganisms including bacteria and fungi. However, the mineralogy composition of such nanostructures would depend on the physico-chemical properties of the precipitating solution (chemistry, pH, salinity, etc.,) and on the type of microorganism involved in the precipitation.

The aqueous culture medium used in these experiments is saturated with respect to vivianite and siderite (Table 1), there is a tendency toward their abiotic precipitation. The calculated saturation indexes (Table 1) for vivianite and siderite indicate that they should have been abiotically precipitated in the solution, in the absence of bacteria. However, no mineral precipitation was observed in the control experiments (without and with non-viable cells), while in living culture experiments (with active cells) vivianite and siderite precipitated. These data confirm that an aqueous solution saturated with certain mineral phase(s) does not imply abiotic precipitation with respect to those minerals, but it depends on their precipitation kinetics (Morse, 1983). Therefore, vivianite and siderite precipitation can be attributed to the presence of living *T. lapidicaptus* cells which are capable of overcoming the kinetic barriers for mineral precipitation, i.e., reducing the activation energy barriers. The metabolic activity of the bacteria is very important because it supplies the ions necessary for the formation of minerals, PO_4_^3−^ for phosphates and CO_3_^2−^ for carbonates. Additionally, the appropriate microenvironment around bacterial cells and EPS (increase pH and/or ionic concentration) is created for mineral precipitation. In fact, an increase in the pH from 6 to 7.8 in the cultures with active cells was measured. No mineralization was observed in the control experiments where no pH alteration was detected.

Bacteria induce mineral precipitation by concentrating ions (e.g., Ca, Fe, Mg, CO_3_^2−^, PO_3_^4−^, ${\text{NH}}_{4}^{+}$) and changing the pH in the microenvironment surrounding their cells (Ehrlich, 2002; van Lith et al., 2003; Sánchez-Román et al., 2011). Bacterial cells act as a template for mineral nucleation by adsorbing ions around the cellular surface membrane or cell wall (Schultze-Lam et al., 1996; Bosak and Newman, 2003). This process does not occur in absence of bacterial activity. Moreover, EPS are considered as important factor for mineral precipitation (Dupraz et al., 2004; Aloisi et al., 2006; Ercole et al., 2007; Bontognali et al., 2008; Krause et al., 2012). The charged cell walls as well as the reactive groups of the EPS provide active interfacial sites for adsorption and complexation of dissolved aqueous metal species, inducing the nucleation and precipitation of minerals by reducing the activation energy barriers (Konhauser, 1998; De Yoreo et al., 2013; Habraken et al., 2013). This results in a mineralized cellular matrix containing detectable concentrations of metallic ions that are not easily re-dissolved (Beveridge and Fyfe, 1985). During bacterial growth experiments using an aqueous medium rich in organic compounds (yeast extract, glucose, succinic anhidryd, cysteine) source for CO_2_, NH_3_ and HPO_3_^2−^, the pH, carbonate and phosphate concentrations increased because of the production of CO_2_, NH_3_, and HPO_3_^2−^ (which hydrate to form CO_3_^2−^, ${\text{NH}}_{4}^{+}$ and PO_4_^3−^) during metabolization of organic compounds.

These changes together with the adsorption of iron ions by *T. lapidicaptus* would lead to local supersaturation gradients around bacterial surfaces and EPS, using these as nucleation sites to induce iron phosphate and carbonate precipitation as previously has been demonstrated for other type of bacteria (Aloisi et al., 2006; Bontognali et al., 2008; Sánchez-Román et al., 2008, 2011, 2014).

It is essential to understand how super-saturation and nucleation develop in our culture experiments. If concentration of ions in solution exceeds the solubility product for a solid mineral phase, precipitation will not occur until a certain degree of supersaturation is achieved (Berner, 1980). The process during which the maximum free energy is attained is known as nucleation and involves the growth of critical crystal nuclei (Jack et al., 1993; Sánchez-Navas et al., 2009). This process is accompanied by a decrease in free energy and is referred to as crystal growth. The presence of living bacteria can promote either process. Here, the surface of the microbial cell and EPS provides a template on which nucleation can occur and overcome kinetic barriers to facilitate mineral precipitation as previously expounded. This reduces the free energy required during the nucleation step and focuses crystal growth because nucleation on the biological template occurs before nucleation in homogeneous solution (Jack et al., 1993). Most types of bacteria are capable of acting as nucleation templates (Beveridge and Fyfe, 1985). Although abiotic precipitation is difficult in natural systems or in sterile laboratory experiments (present work), the presence of bacteria can induce the precipitation of minerals in microenvironments by (1) modifying the conditions of their surrounding environments and/or concentrate ions in the bacterial cell envelope and (2) acting as nucleation sites.

Apparently, siderite and vivianite have many physico-chemical characteristics in common. However, morphological details suggest different nucleation and growth conditions. In most cases, siderite nucleates on the bacterial cell wall and with time develops into a microspherulite (Figures 2A–C, 4A–D); and vivianite nucleates on the bacterial cell and within the EPS on nanocrystals that agglomerate and get alone as elongated (rosette) crystals (Figures 2A–E, 3A–D). On the other hand, PO_4_^3−^ inhibits the precipitation of carbonate minerals (Bouropoulos and Koutsoukos, 2000; Kofina and Kotsoukos, 2005; Morse et al., 2007). Whereas, the presence of phosphate can inhibit the formation of siderite (Fredrickson and Gorby, 1996). Indeed, when phosphate is present, vivianite appears to be the stable end product due to its lower solubility product (Ksp = 10^−36^) (Glasauer et al., 2003). In our cultures, vivianite precipitated first than siderite. Organic phosphates are hydrolysed by phosphatases, which liberate orthophosphate during microbial decomposition of organic material. Locally elevated orthophosphate, excreted during microbial decomposition of organic material (yeast extract), around bacterial cells becomes available together with the inorganic phosphate and soluble iron (Fe^2+^) initially present in the culture medium for the precipitation of vivianite. Hence, the precipitation of Fe-phosphate (vivianite) removes PO_4_^3−^ ions from the solution, leading to the precipitation of Fe-carbonate (siderite). Thus, we propose that vivianite and siderite are authigenic sedimentary minerals that require similar physico-chemical conditions to precipitate and the presence of microorganisms.

### Significance and implications of vivianite and siderite in natural systems

In our aqueous solutions, conditions for the precipitation of Fe-carbonates are created after phosphate precipitation. Thus, we propose that in environments with sufficient phosphate and iron, vivianite will precipitate first than siderite, while in environments with PO_4_^3−^ deficiency siderite will precipitate first. The same phenomenon might be occurring in Rio Tinto subsurface, from where *T. lapidicaptus* has been isolated (Puente-Sánchez et al., 2014a), characterized by the presence of iron sulfide (pyrite) and carbonate (siderite) minerals (Fernández-Remolar et al., 2012). However, Fe-phosphates have not yet been detected there, even though vivianite is considered as an alteration of pre-existing Fe-carbonates or sulfides (Garvin, 1998). This could be due to the presence of abundant dissolve sulfide, which inhibits the formation of vivianite (Postma, 1981; Manning et al., 1999). The reduction of aqueous or embedded sulfate coupled to organic matter oxidation would lead to the formation of H_2_S and carbonate. H_2_S subsequently reacts with iron, and contributes to the formation of FeS_2_ whereas CO_3_^2−^ would react with iron to precipitate carbonate (FeCO_3_). Then, the absence of phosphate minerals in Rio Tinto may be linked to the presence of sulfate (Puente-Sánchez et al., 2014b) and its microbiological transformation to H_2_S with the consequently formation of pyrite. It is probably for this reason that we rarely find vivianite occurring in nature. In fact, it only persists in reducing organic-rich environments (lakes, deep-sea sediments, swamps, sewage) with low concentrations of sulfate, which results in a high and continuous precipitation of vivianite. Our experimental findings provide information that could be used to interpret the role of microorganisms in digenetic mineral processes resulting in phosphate and carbonate formation in natural systems.

On the other hand, supersaturated solutions (e.g., interstitial pore water) cannot serve as reliable predictors for the *in situ* formation of phosphates (Rothe et al., 2014). A gel-like pore structure (Rothe et al., 2014) of a sediment matrix rich in organic matter, in combination with release of soluble phosphorous and iron due to microbial activity, is necessary for vivianite formation in natural systems. In our culture experiments the gel-like pore structure would be the bacterial EPS and cell surfaces which throughout bacterial activity (1) create local microenvironments supersaturated with respect to phosphates and carbonates; and (2) act as templates for mineral nucleation overcoming the kinetic barriers of mineral precipitation. Indeed, similar nanocrystals of phosphate and carbonate to the ones shown in the present study (Figures 2A,E, 4A,B) have been also reported as closely linked to the presence of bacterial cells, EPS and similar mucilaginous structures in modern and ancient environments (Sánchez-Román et al., 2008; Crosby and Bailey, 2012; Cosmidis et al., 2013, 2014; Sánchez-Navas et al., 2013). The preservation of these nanostructures in the geological record may help us to trace microbial processes through geologic time. Therefore, this experimental study (1) may help to understand the formation of ancient iron phosphate (Fife and Mark, 1982; Cook and Shergold, 1986; Simonen, 1986) and carbonate (Veizer et al., 1989; Ohmoto et al., 2004) deposits, and (2) provides potential biosignatures that may be useful to test terrestrial and extraterrestrial habitats for life evidences.

Finally, our experiments demonstrate that *T. lapidicaptus* can cause considerable iron accumulation through biomineralization of phosphate and carbonate, therefore, *T. lapidicaptus* could be considered a good phosphate removing bacterium from anaerobic systems. Furthermore, vivianite and siderite produced by *T. lapidicaptus* could be a good alternative fertilizer of phosphorous and iron. The presence of this bacterium and/or related bacteria in natural environments could explain the formation of vivianite and siderite. The co-precipitation of Fe-phosphate and carbonate in our cultures links the P, C, and Fe cycles during biomineralization.

## Author contributions

MS and FP designed the culture experiments and performed all the laboratory tasks, carried out the culture experiments. MS wrote the first draft of the manuscript. FP, VP, and RA assisted in preparing the manuscript, all authors read and approved the final version.

### Conflict of interest statement

The authors declare that the research was conducted in the absence of any commercial or financial relationships that could be construed as a potential conflict of interest.
